# 4-Chloro-2-[(4-chloro­benzyl­idene)amino]­phenol

**DOI:** 10.1107/S1600536812019770

**Published:** 2012-05-12

**Authors:** Kürşat Efil, Fatih Şen, Yunus Bekdemir, Orhan Büyükgüngör

**Affiliations:** a Ondokuz Mayıs University, Arts and Sciences Faculty, Department of Chemistry, 55139 Samsun, Turkey; b Kilis 7 Aralık University, Vocational High School of Health Services, Department of Opticianry, 79000 Kilis, Turkey; c Ondokuz Mayıs University, Arts and Sciences Faculty, Department of Physics, 55139 Samsun, Turkey

## Abstract

In the title Schiff base compound, C_13_H_9_Cl_2_NO, the mol­ecule displays an *E* conformation about the imine C=N double bond, with a dihedral angle of 8.09 (11)° between the two benzene rings. In the crystal, mol­ecules are linked by a single O—H⋯O hydrogen bond, giving one-dimensional chains which extend along (100).

## Related literature

For related Schiff base compounds and applications, see: Asiri & Khan (2010 ▶); Bekircan *et al.* (2006 ▶); Faridbod *et al.* (2008 ▶); Fun *et al.* (2009 ▶); Ghanwate *et al.* (2008 ▶); Jarrahpour *et al.* (2007 ▶); Layer (1963 ▶); Shi *et al.* (2007 ▶); Zhao *et al.* (2010 ▶). For related structures, see: Xu *et al.* (2009 ▶); Zhou *et al.* (2009 ▶).
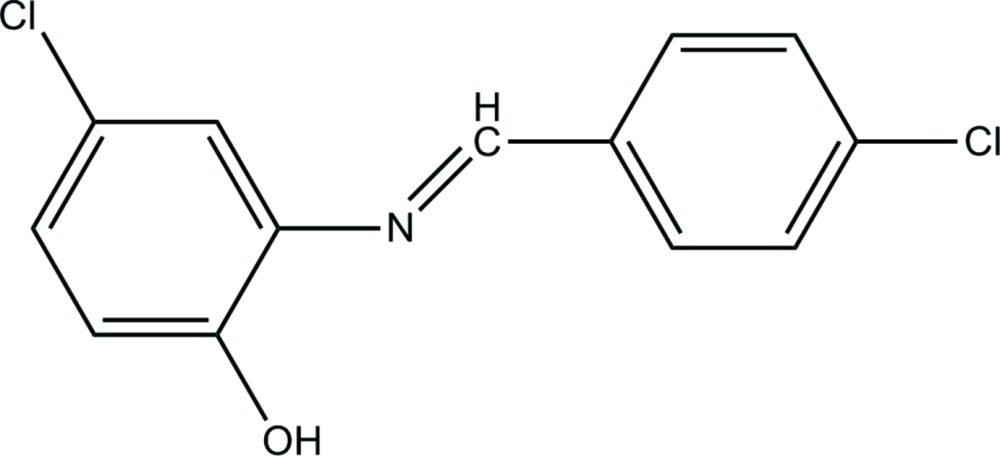


## Experimental

### 

#### Crystal data

C_13_H_9_Cl_2_NO
*M* *_r_* = 266.11
Orthorhombic, 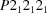

*a* = 4.6615 (2) Å
*b* = 10.5375 (5) Å
*c* = 25.2153 (15) Å
*V* = 1238.59 (11) Å^3^
*Z* = 4
Mo *K*α radiation
μ = 0.51 mm^−1^
*T* = 296 K
0.53 × 0.41 × 0.31 mm

#### Data collection

Stoe IPDS 2 Image-Plate diffractometer
Absorption correction: integration (*X-RED32*; Stoe & Cie, 2002 ▶) *T*_min_ = 0.815, *T*_max_ = 0.882
2562 measured reflections
2562 independent reflections
1910 reflections with *I* > 2σ(*I*)
*R*_int_ = 0.037

#### Refinement

*R*[*F* ^2^ > 2σ(*F* ^2^)] = 0.033
*wR*(*F* ^2^) = 0.082
*S* = 0.93
2562 reflections
159 parameters
H atoms treated by a mixture of independent and constrained refinement
Δρ_max_ = 0.21 e Å^−3^
Δρ_min_ = −0.24 e Å^−3^
Absolute structure: Flack (1983 ▶), 1024 Friedel pairs
Flack parameter: 0.01 (7)

### 

Data collection: *X-AREA* (Stoe & Cie, 2002 ▶); cell refinement: *X-AREA*; data reduction: *X-RED32* (Stoe & Cie, 2002 ▶); program(s) used to solve structure: *SHELXS97* (Sheldrick, 2008 ▶); program(s) used to refine structure: *SHELXL97* (Sheldrick, 2008 ▶); molecular graphics: *ORTEP-3 for Windows* (Farrugia, 1997 ▶); software used to prepare material for publication: *WinGX* (Farrugia, 1999 ▶) and *PLATON* (Spek, 2009 ▶).

## Supplementary Material

Crystal structure: contains datablock(s) global, I. DOI: 10.1107/S1600536812019770/zs2205sup1.cif

Structure factors: contains datablock(s) I. DOI: 10.1107/S1600536812019770/zs2205Isup2.hkl

Supplementary material file. DOI: 10.1107/S1600536812019770/zs2205Isup3.cml

Additional supplementary materials:  crystallographic information; 3D view; checkCIF report

## Figures and Tables

**Table 1:** Hydrogen-bond geometry (Å, °)

*D*—H⋯*A*	*D*—H	H⋯*A*	*D*⋯*A*	*D*—H⋯*A*
O1—H1⋯O1^i^	0.86 (3)	2.36 (3)	3.040 (2)	136 (2)

Symmetry code: (i) 
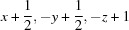
.

**Fig. 1.**
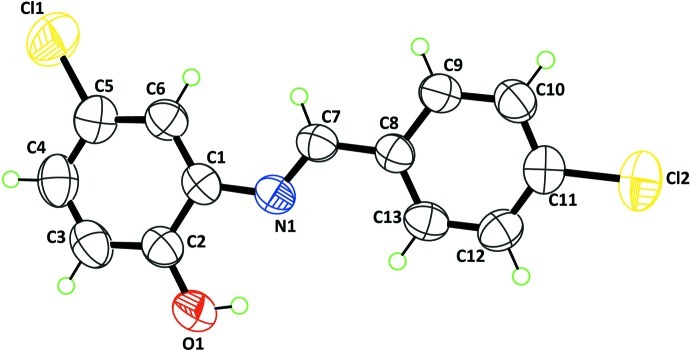
The molecular structure of the title compound showing the atom- numbering scheme, with displacement ellipsoids drawn at the 50% probability level.

**Fig. 2.**
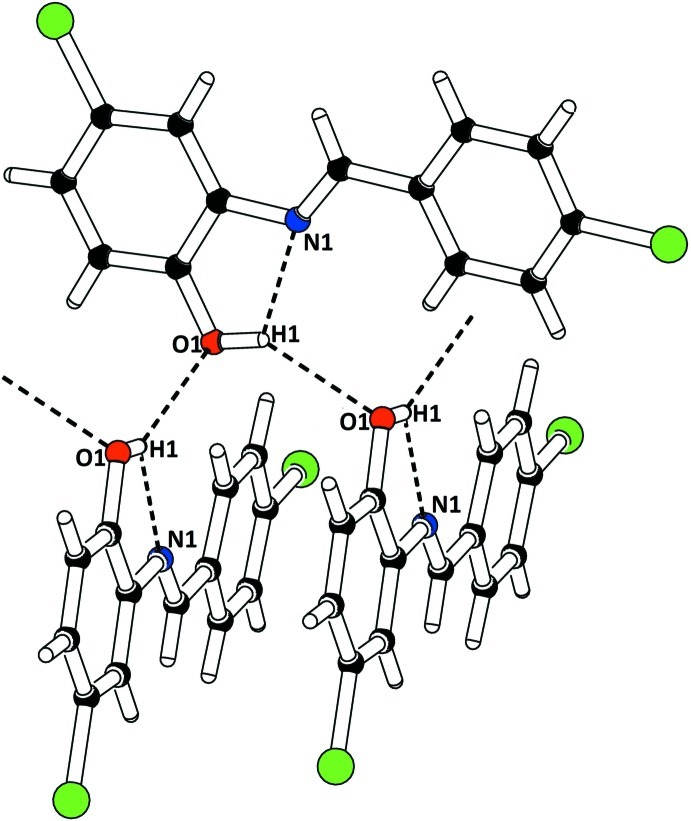
Part of the crystal structure of the title compound, showing the O—H···N and O—H···O interactions as dashed lines.

**Fig. 3.**
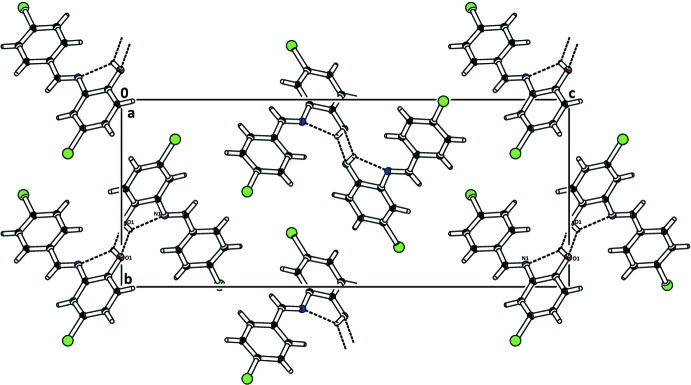
The crystal packing of the title compound viewed along the a axis. The O—H···O and O—H···N interactions are shown as dashed lines.

## supplementary crystallographic information

### Comment

The compounds containing the C═N group, are known as Schiff bases (also imines or azomethines), are generally synthesized by condensation of primary amines with active carbonyl compounds (Asiri & Khan, 2010; Faridbod *et al.*, 2008). The reaction is acid-catalyzed and is usually performed by refluxing the carbonyl compound and amine (Layer, 1963). These compounds show biological activity as antitumor (Zhao, *et al.*, 2010), anticancer (Bekircan, *et al.*, 2006), antifungal (Shi, *et al.*, 2007), antimicrobial (Ghanwate, *et al.*, 2008) or antiviral agents (Jarrahpour, *et al.*, 2007), furthermore they are used as intermediates and ligands in the formation of a complex with some metal ions (Fun *et al.*, 2009). As part of our ongoing study of the structural relationships between the compounds containing Schiff bases, a crystal structure determination of the title compound, C_13_H_9_Cl_2_NO, has been undertaken and the results are presented here.

The structure of the title compound (Fig. 1) is similar to those of analogous derivatives (Xu *et al.*, 2009; Zhou *et al.*, 2009) and displays a *trans* configuration with respect to the imine C═N with a C8—C7—N1—C1 torsion angle of 179.19 (18)°. The molecule is close to planar, as indicated by the dihedral angle between the two benzene rings [8.09 (11)°]. The crystal packing is stabilized by a single intermolecular O—H···O hydrogen-bonding interaction (Table 1, Fig. 2), giving a one-dimensional chain structure which extends down (100) (Fig. 3). An intramolecular O—H···N interaction is also present.

### Experimental

4-Chlorobenzaldehyde (0.141 g; 1 mmol), 2-hydroxy-5-chloroaniline (0.144 g; 1 mmol) and two drops of β-ethoxyethanol as a wetting solvent were mixed in a beaker and then exposed to microwaves in an oven (360 W). It was observed that the reaction was completed within 2 minutes (thin layer chromatography). The resulting solid product was washed with cold ethanol and recrystallized from ethanol, giving the title compound. Yield: 92%, m.p. 396–398 °K.

### Refinement

The H-atom of the hydroxy group was located from a difference-Fourier map and both positional and isotropic displacement paramenters were refined. Other H-atoms were positioned geometrically and treated using a riding model, with C—H = 0.93 Å and with the displacement parameters *U*_iso_(H) = 1.2*U*_eq_(C). The absolute structure factor (Flack, 1983), although not significant in this structure, was determined as 0.01 (7), using 1028 Friedel pairs.

### Crystal data

**Table:**

C_13_H_9_Cl_2_NO	*D*_x_ = 1.427 Mg m^−^^3^
*M**_r_* = 266.11	Melting point = 396–398 K
Orthorhombic, *P*2_1_2_1_2_1_	Mo *K*α radiation, λ = 0.71073 Å
Hall symbol: P 2ac 2ab	Cell parameters from 16508 reflections
*a* = 4.6615 (2) Å	θ = 1.6–27.2°
*b* = 10.5375 (5) Å	µ = 0.51 mm^−^^1^
*c* = 25.2153 (15) Å	*T* = 296 K
*V* = 1238.59 (11) Å^3^	Prism, brown
*Z* = 4	0.53 × 0.41 × 0.31 mm
*F*(000) = 544	

### Data collection

**Table:**

Stoe IPDS 2 CCD diffractometer	2562 independent reflections
Radiation source: fine-focus sealed tube	1910 reflections with *I* > 2σ(*I*)
Graphite monochromator	*R*_int_ = 0.037
rotation method scans	θ_max_ = 26.5°, θ_min_ = 1.6°
Absorption correction: integration (*X-RED32*; Stoe & Cie, 2002)	*h* = −5→5
*T*_min_ = 0.815, *T*_max_ = 0.882	*k* = −13→13
2562 measured reflections	*l* = −31→31

### Refinement

**Table:**

Refinement on *F*^2^	Secondary atom site location: difference Fourier map
Least-squares matrix: full	Hydrogen site location: inferred from neighbouring sites
*R*[*F*^2^ > 2σ(*F*^2^)] = 0.033	H atoms treated by a mixture of independent and constrained refinement
*wR*(*F*^2^) = 0.082	*w* = 1/[σ^2^(*F*_o_^2^) + (0.0496*P*)^2^] where *P* = (*F*_o_^2^ + 2*F*_c_^2^)/3
*S* = 0.93	(Δ/σ)_max_ < 0.001
2562 reflections	Δρ_max_ = 0.21 e Å^−^^3^
159 parameters	Δρ_min_ = −0.24 e Å^−^^3^
0 restraints	Absolute structure: Flack (1983), 1024 Friedel pairs
Primary atom site location: structure-invariant direct methods	Flack parameter: 0.01 (7)

### Special details

**Table:**

Experimental. IR: 3071, 2911, 1626 (C=N),1586, 1568, 1478, 1423, 1369, 1271, 1238, 1194, 1154, 1082, 1009, 909, 856, 812, 696, 607 cm^-1^. 1H NMR (CDCl_3_): δ 8.66 (s, 1H, N=CH), 7.91 (d, J = 8.4 Hz, 2H, Ar), 7.54 (d, J = 8.4 Hz, 2H, Ar), 7.34 (s, 1H, Ar), 7.24 (d, J = 8.6 Hz, 1H, Ar), 7.02 (d, J = 8.6 Hz, 1H, Ar). ^13^CNMR (CDCl_3_): δ 156.75 (N=C), 150.94, 138.21, 135.86, 133.87, 130.06, 129.28, 128.77, 125.07, 116.19. Elemental Anal. Calcd for C_13_H_9_Cl_2_NO: C, 58,67; H, 3,41; N, 5,26. Found: C, 57.74; H, 3.42; N, 5.26%.
Geometry. All e.s.d.'s (except the e.s.d. in the dihedral angle between two l.s. planes) are estimated using the full covariance matrix. The cell e.s.d.'s are taken into account individually in the estimation of e.s.d.'s in distances, angles and torsion angles; correlations between e.s.d.'s in cell parameters are only used when they are defined by crystal symmetry. An approximate (isotropic) treatment of cell e.s.d.'s is used for estimating e.s.d.'s involving l.s. planes.
Refinement. Refinement of *F*^2^ against ALL reflections. The weighted *R*-factor *wR* and goodness of fit *S* are based on *F*^2^, conventional *R*-factors *R* are based on *F*, with *F* set to zero for negative *F*^2^. The threshold expression of *F*^2^ > σ(*F*^2^) is used only for calculating *R*-factors(gt) *etc*. and is not relevant to the choice of reflections for refinement. *R*-factors based on *F*^2^ are statistically about twice as large as those based on *F*, and *R*- factors based on ALL data will be even larger.

### Fractional atomic coordinates and isotropic or equivalent isotropic displacement parameters (Å^2^)

**Table:**

	*x*	*y*	*z*	*U*_iso_*/*U*_eq_	
C1	−0.1216 (4)	0.47092 (19)	0.57988 (7)	0.0537 (5)	
C2	−0.2380 (5)	0.4456 (2)	0.53004 (8)	0.0616 (6)	
C3	−0.4413 (6)	0.5255 (3)	0.50863 (9)	0.0769 (6)	
H3	−0.5168	0.5082	0.4753	0.092*	
C4	−0.5333 (6)	0.6303 (2)	0.53591 (9)	0.0760 (7)	
H4	−0.6710	0.6839	0.5213	0.091*	
C5	−0.4197 (6)	0.6552 (2)	0.58495 (9)	0.0713 (6)	
C6	−0.2162 (5)	0.5778 (2)	0.60712 (8)	0.0628 (6)	
H6	−0.1414	0.5968	0.6404	0.075*	
C7	0.1973 (5)	0.3864 (2)	0.64196 (7)	0.0561 (5)	
H7	0.1415	0.4505	0.6651	0.067*	
C8	0.4106 (4)	0.29397 (18)	0.65979 (7)	0.0523 (4)	
C9	0.5145 (5)	0.2984 (2)	0.71173 (8)	0.0653 (6)	
H9	0.4480	0.3612	0.7346	0.078*	
C10	0.7136 (5)	0.2117 (2)	0.72989 (8)	0.0663 (6)	
H10	0.7827	0.2163	0.7644	0.080*	
C11	0.8069 (5)	0.1196 (2)	0.69631 (8)	0.0606 (5)	
C12	0.7106 (5)	0.1122 (2)	0.64475 (8)	0.0634 (6)	
H12	0.7792	0.0493	0.6222	0.076*	
C13	0.5123 (5)	0.19875 (19)	0.62699 (7)	0.0605 (5)	
H13	0.4452	0.1933	0.5923	0.073*	
N1	0.0866 (4)	0.38240 (15)	0.59657 (6)	0.0554 (4)	
O1	−0.1527 (4)	0.34241 (18)	0.50249 (6)	0.0813 (5)	
Cl1	−0.5403 (2)	0.78790 (6)	0.61993 (3)	0.1114 (3)	
Cl2	1.05363 (14)	0.00872 (6)	0.71919 (3)	0.0847 (2)	
H1	−0.021 (6)	0.303 (2)	0.5193 (9)	0.088 (9)*	

### Atomic displacement parameters (Å^2^)

**Table:**

	*U*^11^	*U*^22^	*U*^33^	*U*^12^	*U*^13^	*U*^23^
C1	0.0469 (11)	0.0618 (12)	0.0525 (10)	−0.0110 (10)	0.0007 (9)	0.0069 (9)
C2	0.0571 (13)	0.0738 (14)	0.0538 (11)	−0.0057 (11)	0.0013 (10)	0.0010 (10)
C3	0.0699 (14)	0.1008 (17)	0.0599 (12)	−0.0012 (16)	−0.0072 (12)	0.0118 (12)
C4	0.0673 (15)	0.0796 (15)	0.0810 (15)	0.0034 (14)	0.0001 (14)	0.0274 (12)
C5	0.0709 (14)	0.0595 (12)	0.0834 (14)	−0.0046 (13)	0.0028 (13)	0.0115 (11)
C6	0.0658 (14)	0.0580 (12)	0.0646 (12)	−0.0084 (11)	−0.0036 (12)	−0.0010 (10)
C7	0.0544 (12)	0.0618 (12)	0.0522 (10)	−0.0065 (11)	0.0008 (10)	−0.0054 (9)
C8	0.0465 (11)	0.0578 (10)	0.0526 (9)	−0.0073 (10)	−0.0004 (9)	−0.0009 (9)
C9	0.0635 (14)	0.0747 (13)	0.0578 (11)	0.0046 (12)	−0.0047 (11)	−0.0110 (10)
C10	0.0597 (13)	0.0806 (15)	0.0586 (11)	0.0007 (13)	−0.0060 (11)	−0.0009 (11)
C11	0.0520 (12)	0.0595 (12)	0.0704 (13)	−0.0066 (10)	0.0059 (11)	0.0149 (10)
C12	0.0680 (14)	0.0581 (12)	0.0642 (12)	−0.0003 (11)	0.0127 (11)	−0.0008 (10)
C13	0.0696 (14)	0.0629 (11)	0.0490 (10)	−0.0084 (12)	0.0022 (10)	0.0001 (9)
N1	0.0556 (10)	0.0584 (9)	0.0521 (9)	−0.0072 (9)	−0.0013 (8)	0.0006 (7)
O1	0.0827 (12)	0.1033 (13)	0.0580 (8)	0.0137 (10)	−0.0125 (9)	−0.0136 (9)
Cl1	0.1263 (7)	0.0703 (4)	0.1377 (6)	0.0207 (5)	−0.0080 (6)	−0.0116 (4)
Cl2	0.0697 (3)	0.0794 (4)	0.1051 (4)	0.0080 (4)	0.0068 (3)	0.0285 (3)

### Geometric parameters (Å, º)

**Table:**

C1—C6	1.391 (3)	C7—H7	0.9300
C1—C2	1.395 (3)	C8—C13	1.384 (3)
C1—N1	1.411 (3)	C8—C9	1.397 (3)
C2—O1	1.350 (3)	C9—C10	1.381 (3)
C2—C3	1.378 (3)	C9—H9	0.9300
C3—C4	1.370 (3)	C10—C11	1.359 (3)
C3—H3	0.9300	C10—H10	0.9300
C4—C5	1.371 (3)	C11—C12	1.378 (3)
C4—H4	0.9300	C11—Cl2	1.738 (2)
C5—C6	1.370 (3)	C12—C13	1.374 (3)
C5—Cl1	1.746 (2)	C12—H12	0.9300
C6—H6	0.9300	C13—H13	0.9300
C7—N1	1.256 (2)	O1—H1	0.86 (3)
C7—C8	1.462 (3)		
			
C6—C1—C2	118.48 (19)	C13—C8—C9	117.77 (19)
C6—C1—N1	127.32 (18)	C13—C8—C7	122.12 (17)
C2—C1—N1	114.19 (18)	C9—C8—C7	120.10 (18)
O1—C2—C3	119.6 (2)	C10—C9—C8	121.4 (2)
O1—C2—C1	120.2 (2)	C10—C9—H9	119.3
C3—C2—C1	120.2 (2)	C8—C9—H9	119.3
C4—C3—C2	120.8 (2)	C11—C10—C9	118.75 (19)
C4—C3—H3	119.6	C11—C10—H10	120.6
C2—C3—H3	119.6	C9—C10—H10	120.6
C3—C4—C5	119.1 (2)	C10—C11—C12	121.6 (2)
C3—C4—H4	120.5	C10—C11—Cl2	119.01 (16)
C5—C4—H4	120.5	C12—C11—Cl2	119.37 (18)
C6—C5—C4	121.5 (2)	C13—C12—C11	119.3 (2)
C6—C5—Cl1	119.56 (19)	C13—C12—H12	120.4
C4—C5—Cl1	119.0 (2)	C11—C12—H12	120.4
C5—C6—C1	120.0 (2)	C12—C13—C8	121.13 (18)
C5—C6—H6	120.0	C12—C13—H13	119.4
C1—C6—H6	120.0	C8—C13—H13	119.4
N1—C7—C8	122.50 (19)	C7—N1—C1	122.18 (17)
N1—C7—H7	118.8	C2—O1—H1	110.6 (17)
C8—C7—H7	118.8		
			
C6—C1—C2—O1	−179.7 (2)	N1—C7—C8—C9	−176.3 (2)
N1—C1—C2—O1	1.0 (3)	C13—C8—C9—C10	0.5 (3)
C6—C1—C2—C3	0.2 (3)	C7—C8—C9—C10	179.33 (19)
N1—C1—C2—C3	−179.2 (2)	C8—C9—C10—C11	−0.7 (3)
O1—C2—C3—C4	179.5 (2)	C9—C10—C11—C12	0.9 (3)
C1—C2—C3—C4	−0.4 (4)	C9—C10—C11—Cl2	−179.06 (16)
C2—C3—C4—C5	0.2 (4)	C10—C11—C12—C13	−1.0 (3)
C3—C4—C5—C6	0.2 (4)	Cl2—C11—C12—C13	179.01 (16)
C3—C4—C5—Cl1	−179.31 (19)	C11—C12—C13—C8	0.8 (3)
C4—C5—C6—C1	−0.3 (4)	C9—C8—C13—C12	−0.6 (3)
Cl1—C5—C6—C1	179.12 (17)	C7—C8—C13—C12	−179.35 (19)
C2—C1—C6—C5	0.2 (3)	C8—C7—N1—C1	179.19 (18)
N1—C1—C6—C5	179.4 (2)	C6—C1—N1—C7	5.9 (3)
N1—C7—C8—C13	2.5 (3)	C2—C1—N1—C7	−174.84 (19)

### Hydrogen-bond geometry (Å, º)

**Table:**

*D*—H···*A*	*D*—H	H···*A*	*D*···*A*	*D*—H···*A*
O1—H1···N1	0.86 (3)	2.18 (2)	2.655 (2)	115 (2)
O1—H1···O1^i^	0.86 (3)	2.36 (3)	3.040 (2)	136 (2)

Symmetry code: (i) *x*+1/2, −*y*+1/2, −*z*+1.
